# First Molecular Evidence of *Ixodiphagus hookeri* (Hymenoptera: Encyrtidae) in *Ixodes ricinus* and *Haemaphysalis concinna* (Acari: Ixodida) Ticks from Inland and Coastal Areas of the Balkan Peninsula

**DOI:** 10.3390/pathogens14070652

**Published:** 2025-07-01

**Authors:** Veronika Blažeková, Michal Stanko, Dana Zubriková, Lucia Vargová, Klaudia Mária Švirlochová, Bronislava Víchová

**Affiliations:** 1 Institute of Parasitology, Slovak Academy of Sciences, Hlinkova 3, 040 01 Košice, Slovakia; sirotnakova@saske.sk (V.B.); stankom@saske.sk (M.S.); zubrikova@saske.sk (D.Z.); lvargova@saske.sk (L.V.); svirlochova@saske.sk (K.M.Š.); 2 Institute of Zoology, Slovak Academy of Sciences, Dúbravská cesta 9, 845 06 Bratislava, Slovakia; 3 Department of Epizootiology, Parasitology and Protection of One Health, University of Veterinary Medicine and Pharmacy in Košice, Komenského 73, 041 81 Košice, Slovakia

**Keywords:** Balkan, Croatia, Bulgaria, ticks, Ixodes, wasps, Ixodiphagus

## Abstract

*Ixodiphagus hookeri* (Howard, 1907) (Hymenoptera: Encyrtidae), a hyperparasitic wasp that parasitizes hard ticks, has been documented in various parts of Europe; however, data on its presence in southeastern regions has been lacking. This study provides the first molecular evidence of *I. hookeri* in ticks from the coastal areas of the Balkan Peninsula, specifically Croatia and Bulgaria. A total of 1043 questing ticks were collected between 2011 and 2013 across 15 locations. Molecular screening revealed *I. hookeri* DNA in *Ixodes ricinus* (Linnaeus, 1758) (Acari: Ixodidae) nymphs from inland Croatia (overall prevalence: 18.72%) and in *Haemaphysalis concinna* (Koch, 1844) (Acari: Ixodidae) nymphs and larvae from coastal Bulgaria (prevalence: 17.2%). All *I. hookeri*-positive samples were co-infected with *Wolbachia* spp. (Rickettsiales: Anaplasmataceae). This detection marks the southernmost record of *I. hookeri* in Central Europe, expanding its known range to the Balkan Peninsula and supporting its relevance as a potential natural enemy in integrated tick management strategies.

## 1. Introduction

Ticks (order Ixodida) are hematophagous ectoparasites of major medical and veterinary importance due to their role as vectors of numerous pathogens, including viruses, bacteria, and protozoa [1]. The transmission of pathogens through tick bites leads to diseases that can affect multiple organ systems and present with symptoms ranging from mild flu-like illness to severe neurological, cardiac, or hematological complications. Tick-borne diseases (TBDs) such as Lyme borreliosis, tick-borne encephalitis, anaplasmosis, and babesiosis have been increasingly reported across Europe, driven in part by climatic and environmental changes, increased wildlife populations, and growing human mobility [2,3]. 

The Balkan Peninsula, with its complex topography, diverse habitats, and transitional climate zones, is particularly rich in tick species. At least 32 species across five genera, including *Ixodes*, *Rhipicephalus*, *Dermacentor*, *Hyalomma*, and *Haemaphysalis*, have been documented in this region, many of which are competent vectors of zoonotic pathogens [4,5,6]. Several bacterial pathogens have been detected in ticks across the Balkan region, including the *Borrelia burgdorferi* sensu lato complex—the widely distributed causative agent of Lyme borreliosis–as well as *Anaplasma phagocytophilum*, *Rickettsia* spp. (notably those from the spotted fever group), *Coxiella burnetii*, and *Ehrlichia* spp., all of which have been reported in ticks from Albania, Croatia, Serbia, Montenegro, Bosnia and Herzegovina, and other countries in the region [5,7]. The re-emergence of the spotted fever group has been documented, with clinical cases reported in countries such as Serbia, Bulgaria, and Croatia, highlighting their growing public health importance [5,7,8,9].

Protozoan pathogens such as *Babesia* spp. and *Theileria* spp. are also prevalent in the region, affecting domestic animals and humans. Viral pathogens, including tick-borne encephalitis virus (TBEV) and Crimean–Congo hemorrhagic fever virus (CCHFV), have been reported with increasing frequency [5]. The coastal regions along the Mediterranean and Black Seas represent key hotspots for human–vector interactions, especially during the summer months, when millions of tourists visit these areas [10,11]. This seasonal influx increases the risk of human exposure to infected ticks, underscoring the urgent need for effective and ecologically sustainable tick control strategies.

**Figure 1:**
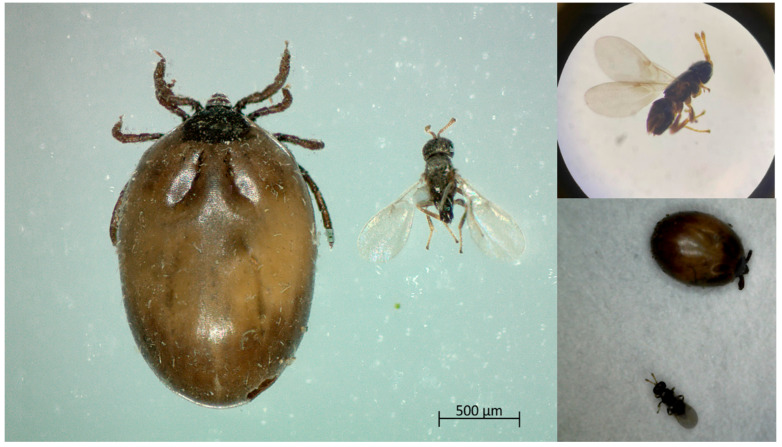
Adult *Ixodiphagus hookeri* wasp that emerged from an *Ixodes* ricinus *nymph*. Image captured using a ZEISS Stemi 508 stereomicroscope (ZEISS, Oberkochen, Germany) with an Axiocam ERc 5s camera.

Among emerging biological control options, parasitoid wasps have received attention due to their species-specific life cycles and potential to reduce tick populations in natural settings. *Ixodiphagus hookeri* (Hymenoptera: Encyrtidae) is a cosmopolitan tick parasitoid known to parasitize both hard (Ixodidae) and soft (Argasidae) ticks [12,13,14]. The female wasp oviposits into developing tick stages, primarily nymphs, and its larvae develop internally, ultimately leading to the death of the tick host upon adult emergence (Figure 1) [14,15]. Due to this life cycle, *I. hookeri* has long been considered a promising candidate for the biological suppression of tick populations.

Despite its global distribution, the presence and ecological role of *I. hookeri* remain poorly studied in many parts of Europe, particularly in southeastern regions such as the Balkan Peninsula. While isolated reports of *I. hookeri* have emerged from central and western Europe, including Hungary, Germany, Poland, and Slovakia [13,16,17], data from the Mediterranean and Black Sea coastlines are absent. Environmental factors, tick host availability, and parasitism rates may influence the local distribution of this parasitoid, warranting region-specific investigation [14,17].

Recent advances in molecular diagnostics, particularly the use of species-specific PCR and sequencing of mitochondrial markers such as COI and 16S rRNA, have significantly improved the detection of *I. hookeri* in field-collected ticks [13,17]. These tools have enabled more accurate assessments of host–parasite associations and have uncovered cryptic occurrences of *I. hookeri* in previously undocumented regions.

Given the ongoing public health threat posed by re-emerging TBDs in the Balkans, and the potential of *I. hookeri* as a biological control agent, this study was undertaken to address a knowledge gap more than a decade after the original sample collection.

In this study, we present the molecular evidence of *I. hookeri* parasitizing ticks collected from coastal ecosystems of the Mediterranean and Black Sea regions of Croatia and Bulgaria, within the Balkan Peninsula. This finding not only extends the known distribution range of *I. hookeri* into southeastern Europe but also contributes novel insights into its ecology and potential as a natural regulator of tick populations in regions of significant public health relevance.

## 2. Materials and Methods

### 2.1. Study Area 

Geographically, Croatia encompasses a diverse western Balkan landscape that includes mountain ranges, a long Adriatic coastline, and lowland plains. The karstic limestone mountains form a distinct natural border between the continental interior and the Mediterranean littoral, influencing local climate and vegetation zones [18].

Bulgaria lies at the junction of the temperate and subtropical climate zones, with a highly heterogeneous topography. Elevations range from lowland areas around 200 m a.s.l. to the highest peak in the Balkans, Musala (2925 m a.s.l.). This variation defines five major climatic regions, contributing to significant biodiversity in flora and fauna, including tick populations [19].

### 2.2. Sampling Sites/Tick Collection

To assess the presence of *I. hookeri* in ticks from southern Europe, sampling was conducted at 15 model locations across the Balkan Peninsula—specifically, in Croatia (9 sites) and Bulgaria (6 sites). Tick sampling was performed in both inland and coastal zones, covering a range of climatic conditions, from Mediterranean along the coasts to more continental climates inland.

**Figure 2:**
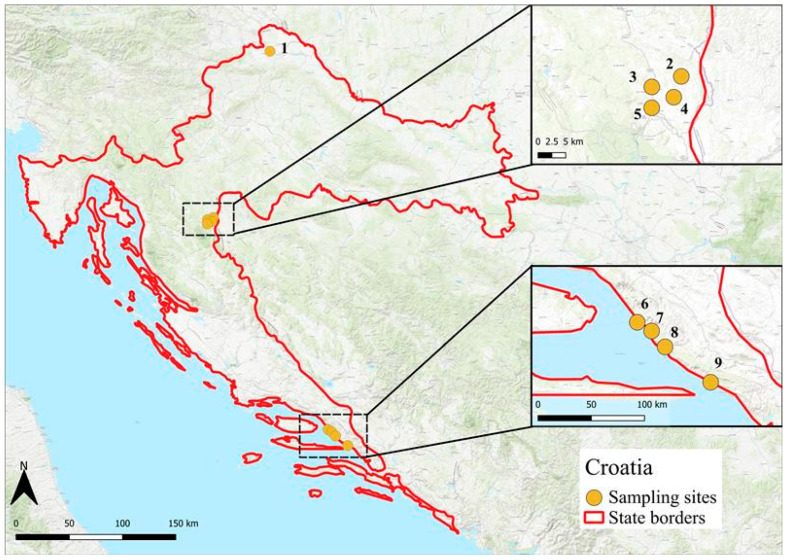
Map of tick collection sites in Croatia (2011). Locations span inland (sites 1–5) and coastal (sites 6–9) zones (north → south: 1. Pušćine, 2. Mašvina, 3. Rakovica, 4. Stara Kršlja, 5. Grabovac, 6. Makarska, 7. Tučepi, 8. Sirena, and 9. Drvenik). The coordinates are listed in the Methods section.

In Croatia, ticks were collected from a total of nine locations (Figure 2) between April and May 2011. These included one site in northern Croatia (Puščine/46°21.384′ N, 16°20.890′ E), four inland sites in the vicinity of Plitvice Lakes National Park (Mašvina/45°1.215′ N, 15°42.227′ E, Rakovica/44°59.775′ N, 15°39.328′ E, Stara Kršlja/45°00.027′ N, 15°40.074′ E, and Grabovac/44°58.279′ N, 15°38.416′ E), and four coastal sites along the Dalmatian coast (Makarska/43°17.122′ N, 17°01.751′ E, Tučepi/43°16.445′ N, 17°02.832′ E, Drvenik/43°09.539′ N, 17°14.428′ E, and Sirena/43°24.344′ N, 16°47.392′ E). 

**Figure 3:**
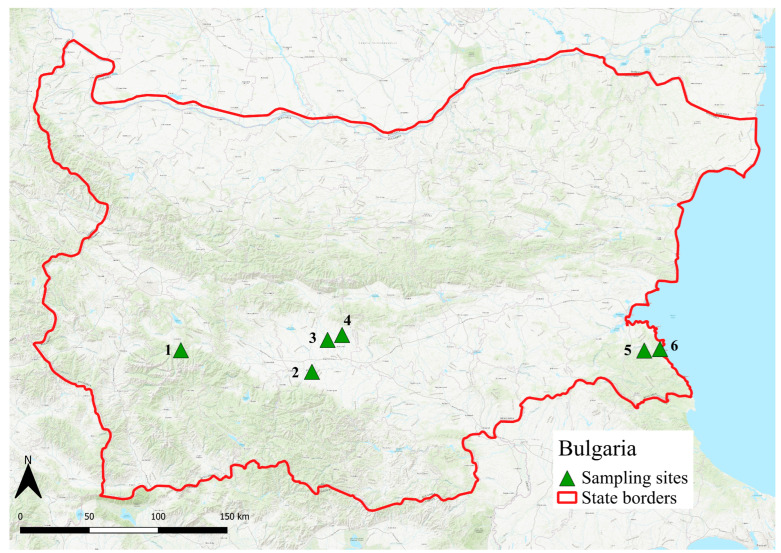
Map of tick collection sites in Bulgaria (2013). The sites include inland regions and coastal Black Sea areas (west →east: 1. Borovec, 2. Plovdiv, 3. Glavatar, 4. Drangovo, 5. Yasna Polyana, 6. Primorsko).

In Bulgaria, tick sampling was performed from late May to early June 2013 at six locations (Figure 3). These included one site in the mountainous region near Rila National Park (Borovec/42°22.704′ N, 24°58.062′ E), three sites in the surroundings of Plovdiv (Plovdiv/42°08.512′ N, 24°47.096′ E, Glavatar/42°20.250′ N, 24°52.816′ E, and Drangovo/42°21.995′ N, 25°01.132′ E,), and two coastal sites on the Black Sea (Yasna Polyana/42°15.753′ N, 27°42.559′ E, and Primorsko/42°17.450′ N, 27°45.505′ E). 

Cartographic outputs were generated using QGIS LTR 3.42 Münster, utilizing the ESRI World Topographic Map as the base map.

Ticks were collected using the standard flagging method, where a 1 × 1 m white cotton cloth was mounted on a wooden pole and dragged across the vegetation [16]. At each sampling site, collections were conducted between 9:00 and 11:00 AM under similar weather conditions. After every 1–2 meters of flagging, the cloth was carefully inspected on both sides for attached ticks. Each field session lasted at least one hour per site. 

All ticks were preserved in 70% ethanol and transported to the laboratory. Ticks were identified to species and developmental stage (and sex, where applicable) using morphological keys [20,21].

### 2.3. DNA Extractions and PCR Analyses

In 2024/2025, the genomic DNA was extracted from questing ticks collected in both Croatia (2011) and Bulgaria (2013) using the alkaline hydrolysis method described by Guy and Stanek [22]. All extracted DNA samples were stored at −20 °C until further processing. To ensure that no DNA degradation occurred despite long-term storage, a subset of randomly selected samples, collected more than a decade ago, was assessed using a NanoPhotometer™ UV/Vis Spectrophotometer (Implen GmbH, Munich, Germany). The high-quality DNA profiles confirmed the long-term stability of the samples, consistent with previous studies demonstrating the effectiveness of ethanol preservation and −20 °C storage for DNA integrity over extended periods [23]. Each DNA sample was initially screened for the presence of the parasitoid wasp by PCR, targeting two genetic markers: a region of the 28S ribosomal RNA (rRNA) gene and a fragment of the mitochondrial cytochrome c oxidase subunit 1 (*cox1*) gene, according to previously published protocols [13,24]. Following this, all samples were examined for the presence of the endosymbiotic bacterium *Wolbachia* spp., according to Zhou et al. [25].

PCR-positive amplicons corresponding to *I. hookeri* were purified using the NucleoSpin^®^ Gel and PCR Cleanup Kit (Macherey-Nagel GmbH & Co., Düren, Germany). The purified products were subsequently subjected to Sanger sequencing in both directions, using the same primers as those employed in the PCR reactions. Sequencing was carried out by Eurofins Genomics (Ebersberg, Germany). Resulting sequences were compared to reference sequences using the NCBI BLAST tool (https://blast.ncbi.nlm.nih.gov/Blast.cgi, accessed on 15 May 2025), and species identification was confirmed based on ≥99% similarity to known *I. hookeri* sequences in the GenBank database.

## 3. Results

A total of 606 ticks were collected in Croatia, comprising 525 *I. ricinus* (136 females, 170 males, and 219 nymphs) and 81 *Rhipicephalus sanguineus* (43 females and 38 males). All samples were molecularly screened for *I. hookeri* and *Wolbachia* spp. DNA. *Ixodiphagus hookeri* DNA was detected exclusively in the nymphs of *I. ricinus*, with an overall parasitism rate of 18.72% (Table 1). Positive detections were confined to four inland localities: Grabovac, Rakovica, Mašvina, and Stara Kršlja. The highest prevalence was observed in Grabovac, where 17.15% (35/204) of nymphs tested positive. In Rakovica, the prevalence was 3.7%, while a single positive specimen was recorded from both Mašvina and Stara Kršlja. All *I. hookeri*-positive samples also harbored *Wolbachia* spp., indicating a 100% co-occurrence of the endosymbiont with the parasitoid wasp.

In Bulgaria, 437 ticks were collected, consisting of 280 *I. ricinus* (52 females, 23 males, and 205 nymphs), 123 *Haemaphysalis concinna* (1 female, 93 nymphs, and 29 larvae), and 34 female *R. sanguineus*. The presence of *I. hookeri* DNA was confirmed in the nymphs of *H. concinna* from the coastal site of Primorsko, with a prevalence of 17.2% (Table 1). In addition, *I. hookeri* DNA was detected in one of three larval pools of *H. concinna* from the same site. A single *H. concinna* nymph collected from Yasna Polyana, a nearby locality, also tested positive.

Nucleotide sequences of the *cox1* and *28S rRNA* genes of *I. hookeri* were obtained from positive *I. ricinus* and *H. concinna* specimens from the investigated sites in Croatia and Bulgaria. The obtained *cox1* sequences were identical in the overlapping region. The *28S* rRNA sequences were also identical with isolates from *I. ricinus* and/or *H. concinna* nymphs from Slovakia (e.g., PP084999-PP085004), as well as with the most frequent GP15 haplotype (MN956813) identified in four different tick species (*H. concinna, R. microplus, I. persulcatus,* and *D. silvarum*) in Russia and Western Africa by [24], except for one isolate (5BHCN/PP760461), obtained from *H. concinna* in Primorsko, Bulgaria, which was slightly different. Sequence alignment using the EMBOSS Needle Pairwise Alignment Tool revealed 97.8% similarity (520/527 nt) between the 5BHCN isolate and the remaining *28S* rRNA sequences from both countries.

All the nucleotide sequences generated in this study were deposited in the GenBank database and are available under the following accession numbers:

*I. hookeri 28S* rRNA (Croatia): PP760504–PP760506; *cox 1* (Croatia): PV707218-PV707224;

*I. hookeri 28S* rRNA (Bulgaria): PP760500, PP760461, PP760453, PP760446.

## 4. Discussion

In the Balkan Peninsula, at least 32 species of ixodid ticks have been documented across five genera, many of which are competent vectors of zoonotic pathogens [5]. Despite this considerable tick diversity and the associated public and veterinary health concerns, data on hyperparasitoids such as *I. hookeri* are limited in this region.

This study provides the first molecular evidence of *I. hookeri* in *I. ricinus* and *H. concinna* ticks from the coastal and inland regions of Croatia and Bulgaria. These findings represent the southernmost confirmed records of the wasps in Central Europe and mark the first documentation of this parasitoid in ticks from the Balkan Peninsula. Before this study, the southern limit of *I. hookeri* in Central Europe was established in Hungary [16], indicating a potential range expansion into the Balkans.

We detected *I. hookeri* in 18.72% of *I. ricinus* nymphs in Croatia, with the highest prevalence observed in Grabovac (17.15%). In Bulgaria, *I. hookeri* DNA was identified in 17.2% of *H. concinna* nymphs and in one larval pool from the Primorsko locality. These parasitism rates are at the upper end of those reported across Europe. Infestation rates in *I. ricinus* nymphs range from 3.2 to 12.5% in western France, from 19.6 to 20% in southwestern France, 8.2% in southern Italy, and from 0.4 to 2.3% in Finland [13,14]. The relatively high rates reported in this study suggest favorable ecological conditions for parasitoid persistence and reproduction in the studied Balkan habitats. All *I. hookeri*-positive ticks carried DNA of *Wolbachia* sp. The co-detection of *Wolbachia* spp. in all *I. hookeri*-positive samples supports previous findings that the endosymbiont is commonly associated with the parasitoid rather than the tick host [12]. While *Wolbachia* is not known to directly infect ticks, its presence may influence the biology of *I. hookeri*, including reproduction and host selection. 

Consistent with previous research, *I. hookeri* was detected mostly in the nymphal stages of *I. ricinus* and *H. concinna*, and only rarely in larvae. However, parasitoids have been observed to overwinter in unfed or engorged larvae and are transferred transstadially to the nymphal stage in several tick species [9,23,24,25,26]. Occasional detections in larvae, such as those found in Bulgarian samples or reported in several other studies [8,13,20,21,22], may suggest earlier oviposition or less common parasitism strategies under certain environmental conditions.

Although *I. hookeri* is known to parasitize mainly immature stages of ticks, its DNA may also be detected in adults. The frequency of infestation in adult ticks may vary by region. In the study by Gaye et al. [13], parasitoid wasp DNA was detected in 3% (28/785) of the analyzed adult hard ticks across six species from western Africa (Côte d’Ivoire and Senegal) and far eastern Europe (Russia): *Rhipicephalus* (*Boophilus*) *microplus*, *Ixodes persulcatus*, *Dermacentor silvarum*, *Haemaphysalis concinna*, *Amblyomma variegatum*, and *Haemaphysalis japonica*. In contrast, Blažeková et al. [13] confirmed the presence of parasitoid DNA in only one adult *Dermacentor reticulatus* male. This was likely due to residual DNA from earlier developmental stages persisting through the tick’s metamorphosis, rather than indicating active parasitism in adulthood. Such sporadic detections should therefore be interpreted with caution, as they may not reflect a current biological interaction but rather a molecular trace of past infection. The ecological dynamics between *I. hookeri*, its tick hosts, and vertebrate reservoirs are complex and influenced by multiple biotic and abiotic factors. Recent studies suggest that the abundance of ungulates, particularly deer, plays a pivotal role in shaping the prevalence of *I. hookeri* in tick populations [26]. In a large-scale Dutch study, parasitism rates reached up to 16% and were positively correlated with deer density and deer-associated pathogens such as *Anaplasma phagocytophilum*, but not with rodent-associated *Borrelia* sp. [27]. These findings indicate that *I. hookeri* may indirectly influence the transmission dynamics of certain pathogens by modulating the structure and abundance of tick hosts.

While *I. hookeri* has been considered a potential biological control agent of ticks, its effectiveness remains debated. The parasitoid’s life cycle is tightly linked to that of its tick hosts and, indirectly, to vertebrate hosts such as deer. In regions with high deer populations, the density of ticks may remain high despite the presence of *I. hookeri*, limiting its potential impact on overall tick numbers [26,27]. Nevertheless, the relatively high parasitism rates observed in this study suggest their potential role in regulating natural tick populations in localized settings with suitable ecological conditions. It is essential to note, however, that the samples analyzed in this study were collected over a decade ago. Screening more recently collected tick specimens could provide insights into potential changes in parasitoid prevalence and distribution over time. To date, *I. hookeri* has been reported in several European countries, including the Czech Republic [28], Ukraine [29], France [12,30], Germany [31,32], the Netherlands [26,33], Finland [34], Italy [14], the United Kingdom, Slovakia [17,24,35,36,37,38], and Hungary [16]. Our findings extend the known distribution of the wasps in southeastern Europe. 

## 5. Conclusions

The relatively high prevalence rates observed, particularly in nymphal stages of *I. ricinus* and *H. concinna*, suggest that local environmental conditions may support stable populations of *I. hookeri*. These findings not only broaden the known distribution of this tick parasitoid but also underscore the need to consider *I. hookeri* as a potentially valuable, yet underexplored, component of integrated tick management strategies.

## Figures and Tables

**Table 1:** Number of examined ticks and prevalence of *I. hookeri* and *Wolbachia* spp. in various tick species from the Balkan Peninsula.

Country	Tick Species	Developmental Stage	Total of Examined Ticks	Prevalence (%) MIR (%)	*Ixodiphagus hookeri*	*Wolbachia* spp.
Croatia	*I. ricinus*	Male	170	Prevalence (%) (no. of positive ticks)	0	0
		Female	136		0	0
		Nymph	219		**18.72 (n = 41)**	**18.72 (n = 41)**
		**Total**	**525**		**7.81 (n = 41)**	**7.81 (n = 41)**
	*R. sanguineus*	Male	38		0	0
		Female	43		0	0
		**Total**	**81**		**0**	**0**
Bulgaria	*I. ricinus*	Male	23	Prevalence (%) (no. of positive ticks)	0	0
		Female	52		0	0
		Nymph	205		0	0
		**Total**	**280**		**0**	**0**
	*H. concinna*	Female	1		0	0
		Nymph	93		**17.20 (n = 16)**	**17.20 (n = 16)**
		**Total**	**94**		**17.02 (n = 16)**	**17.02 (n = 16)**
		Larvae	29	**MIR (%)**	**3.45 (n = 1)**	**3.45 (n = 1)**
	*R. sanguineus*	Female	34	Prevalence (%) (no. of positive ticks)	0	0

## Data Availability

The original contributions presented in the study are included in the article, further inquiries can be directed to the corresponding author/s.
